# Neoadjuvant chemotherapy reduces the levels of HMGB1 and E-cadherin in patients with breast cancer

**DOI:** 10.1038/s41598-023-41836-5

**Published:** 2023-09-08

**Authors:** Qingchang Su, Xin Wang, Rongchen Zhu, Cuicui Liu, Shanping Sun

**Affiliations:** Department of Breast and Thyroid Surgery, Liaocheng People’s Hospital, 67 Dongchangxi Road, Liaocheng, 252000 Shandong Province China

**Keywords:** Diagnostic markers, Prognostic markers

## Abstract

This study investigated the changes in serum tumor marker levels in patients with breast cancer (BC) after neoadjuvant chemotherapy (NACT) and their potential as prognostic factors in NACT. A total of 134 consecutive patients with BC treated at our hospital between January 2019 and December 2021 were retrospectively analyzed. Patients were treated with NACT based on the docetaxel, epirubicin, and cyclophosphamide (TEC) regimen and assessed for marker levels, T cell subsets, and therapeutic outcomes. Receiver operating characteristic (ROC) curves were constructed to evaluate the predictive performance of the markers. Outcome assessments showed that NACT effectively reduced the tumor size, leading to increased complete remission, partial remission, stable disease, and significantly reduced disease progression. Improved immune function has also been observed after NACT. The levels of two (E-cadherin and HMGB1) out of five markers (CA153, CK19, CEA, E-cadherin, and HMGB1) were significantly reduced after NACT before surgery compared with those at admission, suggesting that NACT modulates the levels of biomarkers. ROC analysis revealed that the area under the curve (AUC) of HMGB1 and E-cadherin combination was 0.87 for discrimination of therapeutic response with a sensitivity and specificity of 91.3% and 88.4%, respectively. Serum tumor marker levels were reduced after NACT in patients with BC. The reduction was most prominent for HMGB1, followed by E-cadherin. These biomarkers can be used to predict the therapeutic response to NACT with an AUC of 0.87, thus offering a new tool to monitor treatment progress in NACT for patients with BC.

## Introduction

Breast cancer (BC) has the highest incidence among the three major malignant tumors in women^1^. In recent years, the disease has a tendency to become younger and seriously threatens the lives and health of patients^2^. Neoadjuvant chemotherapy (NACT), widely used in locally advanced breast cancer (BC), is a systemic chemotherapy regimen that reduces the disease focus prior to surgery. It kills invisible metastatic cells to benefit subsequent surgery and radiotherapy, and has been demonstrated to be effective for both middle and advanced BC^3,4^. The occurrence and development of BC are regulated by multiple mechanisms and are closely related to the immune and molecular subtypes^5,6^. The benefits and efficacy of NACT for BC are therefore highly variable among individual patients and are usually evaluated using various methods^7^, such as physical examination, sonography, and molybdenum target X-ray mammography. However, these methods are often complex, time-consuming, and costly^8,9^, and pose a risk of exposure to radiation. Serum tumor markers have been identified to diagnose cancers and monitor responses to treatment, but their clinical usefulness for BC surveillance and monitoring treatment responses has not yet been fully established, particularly in connection with NACT, despite the fact that tumor markers are able to predict the prognosis and/or response to NACT in patients with specific subtypes. For example, a 70-gene signature has been developed to predict the prognosis of HR-positive/HER2-negative and lymph node-negative patients^10^ or women with early stage BC^11^.

During the transformation of human cells from normal to malignant, some cell surface glycoproteins and lipids would change, leading to elevated expression of tumor cell surface-related antigens and secretion of these antigens into body fluids, such as serum. Many of them have been explored as tumor markers for the diagnosis and prognosis of various cancers^12^. For BC, cytokeratin-19 (CK19), high-mobility group box-1 (HMGB1), E-cadherin, carbohydrate antigen 15–3 (CA15-3), 19–9 (CA19-9), 125 (CA125), carcinoembryonic antigen (CEA), tissue polypeptide-specific antigen (TPS), soluble fragment of cytokeratin 19 (CYFRA21-1), and mucin-like carcinoma-associated antigen (MCA) have been reported as predictive and prognostic markers^13–19^. However, these serum tumor markers have not been fully explored for their use in NACT in BC patients in response to therapies.

This study was conducted to evaluate changes in the levels of serum tumor markers and to investigate their potential to predict therapeutic outcomes following NACT in patients with BC.

## Materials and methods

### Subjects

In this retrospective study, we consecutively recruited 134 patients with BC treated at our hospital between January 2019 and December 2021. Patients were included if they were female, aged between 30 and 75 years, diagnosed with primary BC based on the eighth edition of the AJCC Cancer Staging Manual^20^, did not receive any previous BC-related therapy, such as radiotherapy and endocrine therapy, and completed NACT with survival time > 6 months. Patients were excluded if they had a mental disease, were comorbided with severe heart, liver, kidney, and other organ disorders/diseases, had cachexia or very poor health condition. Had infectious diseases, blood system diseases, metastatic tumors, or other gynecological tumors; or they were allergic to chemotherapy drugs, or had other contraindications preventing the use of NACT. Pregnant and lactating women were excluded from the study. To minimize potential bias in patient selection, all patient data were independently collected from the hospital data bases and from clinician records by two investigators. The data were cross-verified in case of possible inconsistence. Patients with incomplete/inconsistent data set were excluded. This study was approved by the institutional ethics committee of Liaocheng People’s Hospital, and informed consent was obtained from all the patients. This investigation was conducted in accordance with the guidelines outlined in the Declaration of Helsinki and with relevant guidelines and regulations.

### Treatment

After baseline assessment at admission, patients were treated with NACT using an intravenous drip of docetaxel (75 mg/m^2^) on day one, epirubicin (80 mg/m^2^), and cyclophosphamide (500 mg/m^2^) on day two (TEC) regimen^21^ for a 3-weekly cycle. After six chemotherapy cycles, patients were subjected to radical surgery if the treatment was effective. All patients received colony-stimulating factor as a supportive therapeutic from day 2.

### Blood sample analysis

Fasting peripheral blood was collected in tubes containing ethylenediaminetetraacetic acid before and after NACT. The serum was separated by centrifugation at 3000 rpm for 10 min at room temperature, aliquoted, and stored at − 80 °C. The HMGB1 concentration was measured using the Sandwich ELISA of Shino-Test (Tokyo, Japan). The samples were added to the wells of microtiter dishes coated with anti-HMGB1 antibody. After incubation for 24 h at 4 °C, the plates were washed and incubated with a second enzyme-labelled antibody for 1 h at room temperature. A colored solution was added for 30 min, and the HMGB1 concentration was spectrophotometrically determined at 450 nm using a standard curve prepared with the kit. CEA, CK19, CA15-3, and E-cadherin levels were measured by enzymatic chemiluminescent immunoassay on an automatic immunoassay analyzer (Cobas e601 module, Roche, Sweden) using enzyme-linked immunosorbent assay (ELISA) kits from CanAg Diagnostics, Sweden and Roche Diagnostics, Sweden, according to the manufacturer’s instructions. Th17, CD4^+^CD25^+^ Tregs, and Treg cells were assessed using a flow cytometer (FACSymphony A1, BD Biosciences, USA). All samples were assayed in triplicates.

### Evaluation of therapeutic effect

All lesions, except bone metastases, were measured using CT, MRI or ultrasonography, and bony lesions were evaluated using bone scintigraphy. Therapeutic outcomes were evaluated according to The Response Evaluation Criteria in Solid Tumors (RECIST)^22^. The responses were classified as complete remission (CR), partial remission (PR), stable disease (SD) or progressive disease (PD). The total effective rate was calculated as (CR + PR)/(total number of patients) × 100%.

### Statistical analysis

All statistical analyses were performed using the IBM SPSS software (version 18.0; IBM, USA). Normality of the data was determined using the Kolmogorov–Smirnov (K-S) test. Normally distributed data are expressed as mean ± standard deviation (SD). Otherwise, the data were represented as M (P25, P75). Numerical data were compared using paired t-tests. Counting data were expressed as percentages and compared using Chi-square or Fisher’s exact tests. To evaluate the ability of serum tumor markers levels to predict therapeutic outcomes following NACT in patients with BC, receiver operating characteristic (ROC) curves, which are based on true- and false positive rates (sensitivity and 1-specificity) of actual therapeutic response and predicted response, were generated as described^23^. Areas under the ROC curve (AUC) were calculated to measure the predictive performance. For single and combination of biomarkers, binary logistic regression was used to obtain the probability, which was used as the predictive value for ROC curve. The confidence intervals (95% CI) for AUC were calculated are described previously^24^. The higher the AUC value, the higher the accuracy of prediction of markers. To plot the ROC curve, data regarding the therapeutic outcomes and predicated responses were imported into SPSS, converted into binary outcomes (positive or negative) to calculate the sensitivity and specificity at each threshold value and then the ROC curves were plotted using the true positive rate against the false positive rate. *P*-value < 0.05 was considered statistically significant.

### Ethical approval

This study was approved by the institutional ethics committee of Liaocheng People’s Hospital.

## Results

### Baseline clinicopathological characteristics

**Figure 1:**
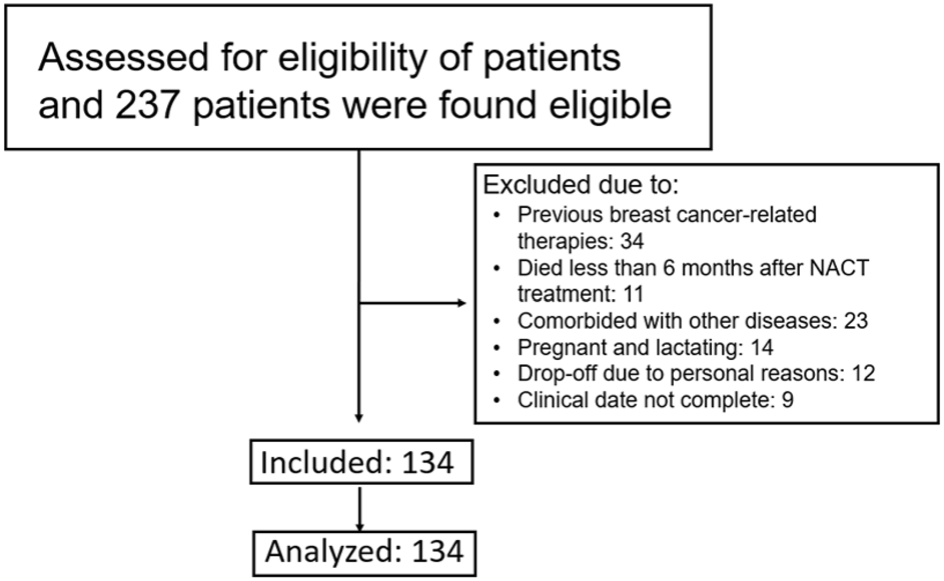
Diagramic chart of patient selection and analysis.

A total of 134 patients were included in this study from 237 eligible patients (Fig. 1). The mean age 41.90 ± 4.43 years. The LD of the lesions was 3.80 ± 1.54 cm, ranging from 0.8 to 8.7 cm. The histological types of tumors included ductal carcinoma in situ (DCIS), invasive ductal carcinoma (IDC), and invasive lobular carcinoma (ILC), and the cancer stage was between TNM stage II and IV, with over 70% being stage II (Table 1). Other baseline data, such as BMI, blood pressure, and renal and liver function tests, are shown in Table 1.

**Table 1:** Baseline clinicopathological characteristics in patients with breast cancer.

Variables	At admission (n = 134)
Age, years	41.90 ± 4.43
Tumor diameter, cm	3.80 ± 1.54
Axillary lymphadenopathy, n (%)	46 (34.2)
Histological type, n (%)	
DCIS	23 (17.1)
IDC	90 (67.1)
ILC	21 (15.7)
TNM stage, n (%)	
II	100 (74.3)
III	29 (21.4)
IV	6 (4.3)
BMI, Kg/m2	32.9 ± 3.3
Blood pressure, mmHg	
Systolic	122.1 ± 7.6
Diastolic	77.8 ± 8.2
Renal function tests	
Creatinine, mg/dL	11.5 ± 3.9
Liver function tests	0.74 ± 0.2
AST, U/L	20.8 ± 4.3
ALT, U/L	27.05 ± 3.1
ALP, U/L	62.8 ± 4.9

DCIS, ductal carcinoma in situ; IDC, invasive ductal carcinoma; ILC, invasive lobular carcinoma; NACT, neoadjuvant chemotherapy.

### NACT effectively reduces tumor size

The outcomes of patients undergoing NACT were assessed at the end of the therapy. The results showed that NACT significantly reduced tumor size (3.80 ± 1.54 vs 1.60 ± 0.64, *P* < 0.01), resulting in a CR of 37.3% and a total response of 70%. PR and SD were 32.9% and 25.7%, respectively (Table 2), demonstrating that NACT is effective in treating BC.

**Table 2:** Therapeutic response of patients with breast cancer after neoadjuvant chemotherapy (NACT) in patients with breast cancer.

Therapeutic responses	After NACT (n = 134)
Tumor diameter, cm	1.60 ± 0.64**
Complete remission, n (%)	50 (37.3)
Partial remission, n (%)	44 (32.9)
Stable disease, n (%)	34 (25.7)
Progression disease, n (%)	5(4.3)
Total, n (%)	94 (70.0)

NACT, neoadjuvant chemotherapy.

**P < 0.01 compared with at admission.

### NACT reduces the level of serum tumor markers

The levels of the five circulating biomarkers were assessed at admission and after NACT. After NACT, there were significant reductions in the levels of HMGB1 and E-cadherin. Compared to that at admission, the overall levels of HMGB1 and E-cadherin were reduced by 62.2% and 55.1%, respectively, at the end of NACT (Table 3, *P* < 0.01). The levels were reduced more remarkably in patients with CR and PR outcomes, and less or not reduced in patients with SD and PD outcomes (Table 3).

**Table 3:** Serum levels of cancer biomarkers at admission and after neoadjuvant chemotherapy (NACT) in patients with breast cancer.

Biomarkers	At admission (n = 134)	After NACT (n = 134)	Therapeutic response			
			CR	PR	SD	PD
CA153, U/mL	26.13 ± 3.15	25.11 ± 2.15	25.89 ± 2.05	25.01 ± 1.75	25.34 ± 2.05	26.21 ± 2.45
CK19, U/mL	2.79 ± 0.94	2.69 ± 0.97	2.56 ± 0.87	2.72 ± 0.87	2.39 ± 0.67	2.79 ± 0.99
CEA, ng/mL	4.43 ± 1.25	4.34 ± 1.21	4.24 ± 1.11	4.32 ± 1.29	4.54 ± 1.31	4.17 ± 1.26
HMGB1, ng/mL	5.77 ± 1.95	2.18 ± 1.15***	0.78 ± 0.25***	1.08 ± 1.05***	3.22 ± 1.75*	5.98 ± 2.15
E-cadherin, ng/mL	1878.41 ± 57.31	842.61 ± 17.31***	242.11 ± 11.21***	472.67 ± 12.41***	1242.21 ± 27.51*	1962.61 ± 62.41

CR, complete remission; PR, partial remission; SD, stable disease; PD, progressive disease.

*, ** and *** *P* < 0.05, 0.01 and 0.001 compared with at admission, respectively.

### HMGB1 and E-cadherin combination has excellent predictive performance on therapeutic response to NACT

**Figure 2:**
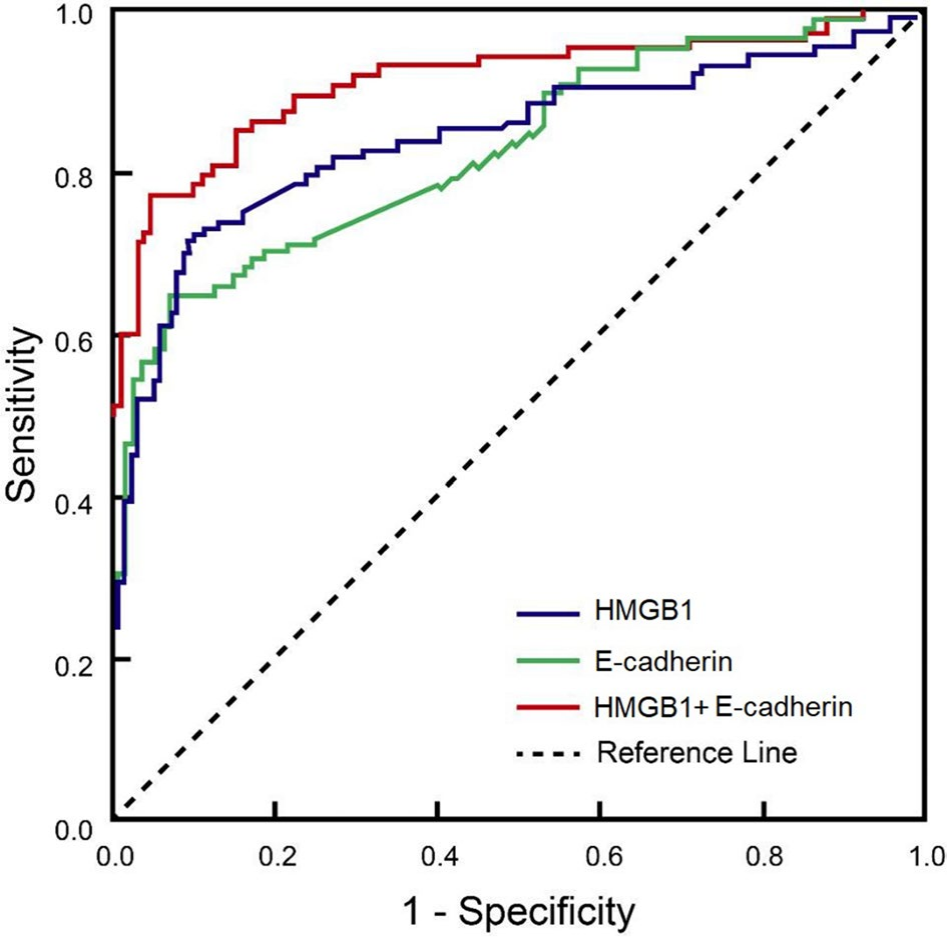
Receiver operating characteristic (ROC) curves of HMGB1 and E-cadherin for the discrimination of a BC with and without favorable therapeutic responses to NACT.

Since the levels of HMGB1 and E-cadherin were significantly reduced after NACT, we analyzed their predictive performance for therapeutic response to NACT. ROC curve analysis showed that the AUCs of HMGB1 and E-cadherin alone were 0.78 and 0.71, slightly lower than 0.80, a value recognized for acceptable predictive marker. On other hand, when the two makers were combined, the AUC reached 0.87 for discrimination of therapeutic response (Fig. 2), indicating that HMGB1 and E-cadherin combination has excellent predictive performance. Furthermore, the sensitivity and specificity of the marker combination were 91% and 88%, respectively, which are better than those of single markers HMGB1 (sensitivity and specificity 82% and 72%) and E-cadherin (sensitivity and specificity 76% and 78%, respectively).

## Discussion

In the present study, we retrospectively analyzed changes in the levels of serum tumor markers before and after NACT and determined the prognostic significance of these markers in patients with BC. Our results showed that NACT had a significant therapeutic effect on BC; the levels of HMGB1 and E-cadherin were reduced after NACT; and the combined use of the two markers could accurately predict the therapeutic response to NACT. Our data suggest that these markers can be used to monitor NACT outcomes in individual patients.

Although NACT has become a routine treatment method for locally advanced BC and could effectively improve the overall efficacy of breast cancer treatment^8^, the assessment of therapeutic response to NACT is mainly based on imaging studies, such as chest and abdominal computed tomography (CT) and bone scintigraphy, which are relatively costly, time consuming, and insensitive. These techniques show only macroscopic changes in tumor volume after several therapy cycles. Serum tumor markers offer promising alternatives for evaluating the therapeutic effects of various methods, such as surgery, chemotherapy, and radiotherapy^25,26^. In this study, we found that TEC regimen-based NACT is effective in BC patients with high CR, PR, and low DP, confirming that NACT is an effective option for downsizing locally advanced BC as anticipated^27^. The CR and PR rates observed in our study were similar to those observed in previous works^28^.

To evaluate the potential of serum biomarkers to measure therapeutic responses, we assessed the levels of the five markers before and after treatment. A significant reduction in the levels was observed in two out of the five markers assessed after NACT, suggesting that NACT modulates the expression and accumulation of these markers. Among the markers evaluated, CA153 is widely used clinically as a serum biomarker for the diagnosis and prognosis of BC. It is used to evaluate chemotherapy efficacy and postoperative outcome^29^. In this study, CA153 levels changed slightly, but not significantly, before and after NACT, suggesting that it could not be used as a predictor of therapeutic response to TEC regimen-based NACT in the treated patient population.

CEA is a broadly proven biomarker for cancers such as colorectal, lung, pancreatic, rectal, and breast cancers^30,31^. Its levels are elevated with aging, cancer, and non-cancer diseases^31^. In the present study, CEA levels were slightly downregulated following NACT. However, although CEA levels were significantly reduced after treatment with taxane and anthracycline-based NACT drugs^32^, it was not useful in predicting BC relapse after curative surgery^33^. Based on the present study, CEA is not a suitable marker for prognosing TEC regimen-based NACT.

CKs are the major structural proteins in epithelial cells, and cells become cancerous when their expression is abnormal^34^. However, the change in CK19 protein is not well documented after NACT. Our data showed that the CK19 levels did not change significantly after NACT. This is consistent with earlier results that CK19 expression did not change significantly in tumor and node biopsies before and after treatment^35^.

HMGB1 has recently been identified as a potential diagnostic biomarker for breast cancer because it may be involved in its development and progression of breast cancer. The sensitivity and specificity of HMGB1 level for the diagnosis of breast cancer were 73.2% and 84.0%, respectively^18^. However, the response to NACT remains controversial. Our assessment showed that serum HMGB1 levels were reduced by over 60% after NACT, suggesting that HMGB1 is likely suppressed by NACT. It is likely that the dynamics of HMGB1 may vary with NACT regimes and cancer subtypes and more studies are needed to elucidate the correlation in various NACT regimens. Previously, HMGB1 levels were found to be higher in non-responders and lower in metastatic breast cancer patients with CR or PR undergoing NACT^36^. However, increased HMGB1 after the initial dose of neoadjuvant epirubicin/docetaxel combination chemotherapy appeared to be associated with better disease-free survival, although the association did not reach significance^37^.

E-cadherin is a transmembrane glycoprotein that functions in calcium-dependent cell-to-cell adhesion^38^. Increased levels of serum E-cadherin are an indication of serious dysfunction of cell surface molecules. Our assessment showed that E-cadherin levels were significantly reduced after NACT, confirming the usefulness of E-cadherin as a predictive marker for NACT. This result is consistent with previous studies in BC^39,40^, but it is in contrast to results obtained in colorectal cancer where E-cadherin is inversely associated with tumor stage^41^.

Although NACT has become a standard clinical option to downsize the tumor for BC-conserving surgery, some BC patients do not respond well to the treatment or even have adverse reactions, resulting in missed surgery due to tumor heterogeneity that causes insensitivity to NACT drugs. Therefore, timely and accurate evaluation of the effect of NACT is important for patients. In this regard, traditional methods such as physical examination, B ultrasound, and X-rays are often not sensitive enough to detect an early response to NACT treatment. The significant changes in the levels of these markers after NACT suggest that they could be used to reflect the therapeutic response in a timely and sensitive manner. ROC analysis showed that the AUCs of HMGB1 and E-cadherin alone were < 0.80, indicating that the use of single markers may not provide adequate predictive accuracy for therapeutic outcomes. However, when the two markers were combined, the AUC was close to 0.9, indicating that the combination had excellent discrimination power for favorable and unfavorable therapeutic responses. To our knowledge, this is the first report demonstrating that the combined use of HMGB1 and E-cadherin is able to provide better predict the treatment response of NACT. For clinical application, HMGB1 and E-cadherin in patients subjected to NACT could be analyzed over the course of treatment in a regular base, for instance, in a weekly basis, as along as the NACT is initiated, and the changes of the two markers be followed along with other pathological response. If the levels of the two markers trend to reduce, this is an indication that the current treatment plant is effective, otherwise, increased or decreased dosing or even ceasing of drugs may be considered.

Although our study demonstrated changes of serum tumor marker level after NACT, the changes, however, are influenced by many factors. Different patient population and breast cancer subtypes are likely response to NACT differently and different biomarkers may be applied to patients with hormone receptor (HR)-positive/human epidermal growth factor 2 (HER2)-negative BC^7^. NACT regimens would also be a major factor influencing the therapeutic outcome as well as the changes in the serum tumor marker level. Although the major chemotherapy regimens include anthracyclines and taxanes, additional drugs such as trastuzumab, pertuzumab and Dual Her2 blockade have been added to NACT^42^. Therefore, the changes in tumor marker levels for various regimens and treatment plans need to be defined since the drugs have different model of actions and will have different impact on the regulation of expression of biomarker.

Our study has some limitations. This was a retrospective investigation at a single center with a relatively small number of patients and heterogeneous subtypes of BC. The NACT in this study was based only on the TEC regimen, and the results obtained might not be applicable to NACT based on other drugs/regimens. In addition, the patients were enrolled mainly based on the availability and were not classified and grouped based on BC subtypes and other demographics such as age. The evaluation of NACT outcomes were made immediately after the completion of NACT and long-term follow-up was not included in the analysis. Analysis of biomarker levels was made only before and after NACT. Prospective studies should include more patients, BC cancer subtypes, NACT regimens and plan, and measurement timepoints to validate the usefulness of these biomarkers, particularly the HMGB1 and E-cadherin combination.

## Conclusions

We examined changes in several serum tumor markers after NACT and the therapeutic response to NACT in patients with BC. We found that HMGB1 and E-cadherin levels were significantly reduced in patients after NACT treatment. This reduction was the greatest in HMGB1 followed by E-cadherin. ROC analysis showed that these biomarkers had prognostic value for NACT and combined use of HMGB1 and E-cadherin had an AUC of 0.87 for discrimination of therapeutic response with the sensitivity and specificity of 91.3% and 88.4%, respectively. Therefore, the combined use of HMGB1 and E-cadherin may offer a new tool for monitoring treatment progress in NACT.

## Data Availability

The datasets used and/or analyzed during the current study are available from the corresponding author on reasonable request.

## Abbreviations

BC: Breast cancer
NACT: Neoadjuvant chemotherapy
TEC: Docetaxel, epirubicin and cyclophosphamide
ROC: Receiver operating characteristic
HMGB1: High mobility group box 1
AUC: Area under curve
CK: Cytokeratin
CA: Carbohydrate antigen
CEA: Carcinoembryonic antigen
TPS: Tissue polypeptide-specific antigen
MCA: Mucin-like carcinoma-associated antigen
ELISA: Enzyme linked immunosorbent assays
RECIST: The response evaluation criteria in solid tumors
LD: Longest diameter
CR: Complete remission
PR: Partial remission
SD: Stable disease
PD: Progression disease
SD: Standard derivation
DCIS: Ductal carcinoma in situ
IDC: Invasive ductal carcinoma
ILC: Invasive lobular carcinoma
TNM: Tumor, node, metastasis
